# Comparative Evaluation of Integrated Waste Heat Utilization Systems for Coal-Fired Power Plants Based on In-Depth Boiler-Turbine Integration and Organic Rankine Cycle

**DOI:** 10.3390/e20020089

**Published:** 2018-01-29

**Authors:** Shengwei Huang, Chengzhou Li, Tianyu Tan, Peng Fu, Ligang Wang, Yongping Yang

**Affiliations:** 1 National Research Center for Thermal Power Engineering and Technology, North China Electric Power University, Changping district, Beijing 102206, China; 2 Shenhua Guohua (Beijing) Electric Power Research Institute, Chaoyang district, Beijing 100025, China; 3 Industrial Process and Energy Systems Engineering, Swiss Federal Institute of Technology in Lausanne, Sion 1951, Switzerland

**Keywords:** coal-fired power plants, waste heat utilization, organic Rankine cycle, exergy analysis, in-depth boiler-turbine integration

## Abstract

To maximize the system-level heat integration, three retrofit concepts of waste heat recovery via organic Rankine cycle (ORC), in-depth boiler-turbine integration, and coupling of both are proposed, analyzed and comprehensively compared in terms of thermodynamic and economic performances. For thermodynamic analysis, exergy analysis is employed with grand composite curves illustrated to identify how the systems are fundamentally and quantitatively improved, and to highlight key processes for system improvement. For economic analysis, annual revenue and investment payback period are calculated based on the estimation of capital investment of each component to identify the economic feasibility and competitiveness of each retrofit concept proposed. The results show that the in-depth boiler-turbine integration achieves a better temperature match of heat flows involved for different fluids and multi-stage air preheating, thus a significant improvement of power output (23.99 MW), which is much larger than that of the system with only ORC (6.49 MW). This is mainly due to the limitation of the ultra-low temperature (from 135 to 75 °C) heat available from the flue gas for ORC. The thermodynamic improvement is mostly contributed by the reduction of exergy destruction within the boiler subsystem, which is eventually converted to mechanical power; while the exergy destruction within the turbine system is almost not changed for the three concepts. The selection of ORC working fluids is performed to maximize the power output. Due to the low-grade heat source, the cycle with R11 offers the largest additional net power generation but is not significantly better than the other preselected working fluids. Economically, the in-depth boiler-turbine integration is the most economic completive solution with a payback period of only 0.78 year. The ORC concept is less attractive for a sole application due to a long payback time (2.26 years). However, by coupling both concepts, a net power output of 26.51 MW and a payback time of almost one year are achieved, which may promote the large-scale production and deployment of ORC with a cost reduction and competitiveness enhancement.

## 1. Introduction

Coal-fired power generation plays a decisive role in the power generation sector in China and is significant to ensure secure electricity supply. Currently, coal-fired power plants contribute around 57% of the total installed power capacity (1.65 billion kW) by the end of 2016. In the last decade, China’s government has imposed a policy of replacing small, non-efficient subcritical power plants with large, highly-efficient supercritical or even ultra-supercritical power plants, which employ very high steam pressure and temperature (over 25 MPa and 600 °C). With such an effort, the specific coal consumption of coal-fired power plants in China has been reduced significantly, for example, 321 g/kWh for plants over 600 MW [1]. From the thermodynamic viewpoint, the above-mentioned reduction of specific coal consumption is mainly because of the development of high-temperature materials, which allow the Rankine cycle to operate at a very high average temperature of heat absorption [2,3,4,5,6,7]. It is also concluded that the system designs of modern large-scale plants still follow those of backward small-scale plants and limited performance improvement has been achieved from system-level heat integration viewpoint [1,8]. The system design of thermal power plants faces a severe requirement of theory and technology innovation to achieve deep energy conservation, considering varying coal quality, complex operating boundary, peak-shaving responsibility and the requirement of ultra-low pollutant emissions [9,10,11,12,13].

Waste heat utilization system (WHUS) is one of the most effective approaches to deeply reduce the exergy losses from boiler and turbine cold-ends, which can result in an improvement of overall system efficiency and a reduction of pollutant emissions [14,15,16,17,18]. There are three main methods with respect to different sources of waste heat and levels of system integration: (1) direct recovery of low-temperature heat from the exhausted flue gas via Organic Rankine Cycle (ORC) [19,20,21] or CO_2_ cycle [22]; (2) utilization of ultra-low-temperature heat in the exhausted steam via heat pump for district heating [23,24]; and (3) deep coupling the turbine and boiler subsystems via low-pressure economizer (LPE) [25,26,27] to enhance the utilization of low- and intermediate-temperature heat within the whole system [28]. The first two approaches bring minimal modifications on the original system layouts and can be easily implemented to existing power plants; however, the third approach is more complicated due to the redesign of the layouts of both boiler and turbine cold-ends. Additionally, due to the locations and temperature levels involved in the first and third approaches, certain synergy may exist and may lead to a further enhanced energy-saving effect.

An ORC system employs organic working fluids with phase change occurring at low temperature, thus can utilize low-temperature waste heat to produce vapor and superheated gas. The ORC technology has attracted increasing attention and has been gradually employed in practical industrial applications. For example, Compana et al. [29] estimated the feasibility of installing ORC units in different industries over 27 EU countries and demonstrated its potential for energy savings and environmental benefits. For practical applications, Cavazaini and Toso [30] conducted a techno-economic feasibility analysis for the integration of a small-scale commercial ORC in a real case study. Pierobon et al. [31] employed a multi-objective optimization approach to identify suitable waste heat recovery technologies for existing and future offshore facilities and concluded that ORC presented better performance than steam Rankine cycle and air bottoming cycle. 

Key issues regarding the design and operation of ORC systems [32] have been investigated, e.g., working-fluid selection [33,34,35], optimal system design for efficiency improvement [35,36] and optimal system control and operation [37]. For working-fluid selection, Hung [38] investigated the effects of different dry fluids on the ORC performance and showed that the irreversibility depended on the type of heat sources. Cavazzini et al. [39] conducted thermodynamic optimization of a sub-critical ORC for heat sources with the temperature level between 80 and 150 °C to choose the optimal fluid from a list of 37 candidates. Pezzuolo et al. [40] performed the fluid selection among 81 possible candidates. For optimal system design and operation, Kermani et al. [35] proposed an approach for integrated design of ORC process and working fluid using PC-SAFT. Branchini et al. [41] performed a systematic thermodynamic comparison of ORC configurations considering various performance indicators. Li et al. [34] provided insights on the system design based on pinch analysis and identified the effects of pinch-point temperature difference (PPTD) in evaporator and condenser of subcritical ORCs on the electricity production cost (EPC). Stoppato [42] thermo-economically analyzed different operating models and offered some guidelines for selecting appropriate incentive of a reference plant sited in Asiago. Further considering the flexibility, safety and less maintenance requirement [32], a basic ORC system is considered for designing an integrated waste heat recovery system for modern coal-fired power plants.

The redesign of the layouts of both boiler and turbine cold-ends, by innovative concepts for system-level heat integration and novel design of heat exchangers working under bad flue gas conditions [43,44,45,46,47], has been emerging in recent years [48,49]. The key idea of such a system-level heat integration is to equip low-temperature economizers (LTE) after or parallel to the air preheat to recover the waste heat from the flue gas, thus suppressing steam extractions for feedwater regeneration [50,51]. In such a way, in-depth recovery of low-temperature waste heat can be achieved. From thermodynamic viewpoint, the fundamental purpose of this approach is introducing the stream splitting to flexibly adjust the heat capacities of the involved heat streams, thus debottlenecking the temperature mismatch of heat integration [52,53]. With the splitting of flue gas, temperature levels of hot and cold streams are better matched with multi-stage air preheating, which reduces the exergy destruction caused by heat transfer and boosts the power output. Several new technologies to recover low-grade heat from humid flue gas, including the latent heat of vapor condensation, have been investigated in [54,55]. Advanced waste heat recovery systems by dividing the air preheating procedure into high-temperature and low-temperature parts were evaluated in [43,56], where the air heating at the low temperature is realized by the exhausted steam extraction and a low-temperature economizer is configured between the electrostatic precipitator and the low-temperature air preheater for feedwater preheating. Two waste heat recovery methods (replacement of regenerative feedwater heaters and implementation of an additional ORC unit) were evaluated in [57] from both thermodynamic and economic viewpoints. These concepts realize better heat cascade utilization at the system level. In our previous work [27], we have also demonstrated that the in-depth interaction between boiler and turbine cold-ends does contribute to a net power output increment of 13.68 MW for a 1000 MW coal-fired power plant.

In this paper, the potential synergies between the ORC and in-depth boiler-turbine integration are investigated to further enhance the system-level heat utilization, since part of the temperature levels are overlapped. The cascade utilization of heat from boiler and turbine cold-ends can be realized by strong heat-exchange interaction among various working fluids (flue gas, extracted steam, feed water, air and the organic working fluids). Three concepts are proposed for comparison purpose: (1) only in-depth boiler-turbine integration (as a follow-up of our previous research [27]); (2) only the ORC; and (3) coupling both the ORC and boiler-turbine interaction. These concepts are analyzed and compared from the energectic, exergetic and economic viewpoints with an emphasis on the third concept. For the ORC, preliminary screening and selection of working fluid for ORC are performed to ensure competitive performance for the available heat source. The paper is organized as follows: In Section 2, the basic power plant and the three proposed concepts are described. Then, in Section 3, the methods employed for working-fluid selection and system evaluation are introduced. Subsequently, in Section 4, the working-fluid selection is first discussed with the influence on system performance and, afterwards, throughout discussion and comparison of all four cases are given. Finally, the conclusions are drawn in Section 5.

## 2. Description of Case Studies

The reference case of the original power plant and the cases with only in-depth boiler-turbine interaction, only ORC, and the coupling of both concepts are introduced with specific specifications, respectively.

### 2.1. The Reference Coal-Fired Power Plant

A typical 1000 MW ultra-supercritical power plant in China is selected as the reference case to quantify the potential of waste heat recovery from the exhausted flue gas. The net power output of the selected power plant is 994 MW with the pressure/temperature of main steam of 26.01 MPa/600 °C and the temperature of reheated steam of 600 °C. The power plant is designed for a bituminous coal with the element analysis of 57.37% carbon, 4.19% hydrogen, 7.57% oxygen, 1.4% nitrogen, 0.87% sulfur and 7.3% moisture. The stream data related to all regenerative heats (RHs) have been listed in Table 1 for the turbine heat rate acceptance (THA) condition, under which the inlet conditions and back pressure of the turbine are nominal with the regenerative feedwater preheaters operating normally. More detailed descriptions of 1000 MW-level coal-fired power plants can be found elsewhere in our publications, e.g., [58,59,60,61,62,63], and will not be repeated here.

### 2.2. The Case with In-Depth Boiler-Turbine Integration

For current regenerative systems of coal-fired power plants, a large amount of steam at various pressure levels are extracted to heat the feedwater and condensate water, which improves the average temperature of heat absorption of the Rankine cycle, thus enhancing the thermodynamic efficiency. However, the working ability of the extracted steam will not be fully realized. The proposed integrated system (modified from [27]) aims at utilizing waste heat from the boiler cold-end to partially heat the feedwater so that some of the steam extraction can be avoided to boost the power generation and system efficiency without introducing additional heat sources.

**Figure 1:**
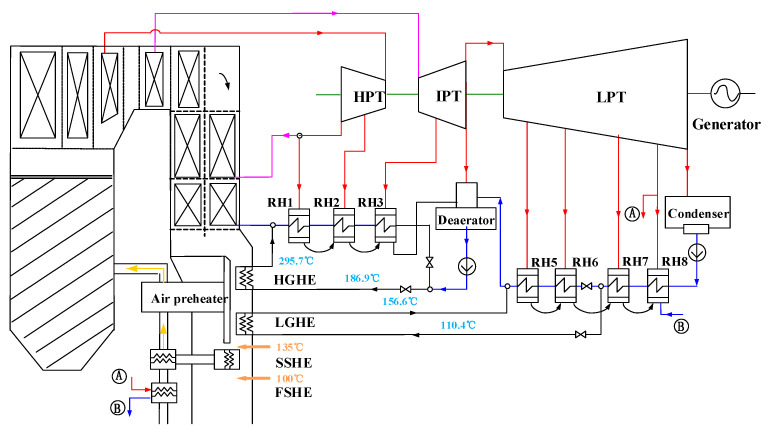
Schematic of the case with in-depth boiler-turbine interaction.

Figure 1 illustrates the configuration of the integrated WHUS system with in-depth boiler-turbine integration. A bypass flue gas duct parallel to the main air-preheater duct is introduced with two-stage successively-configured gas-water heat exchangers. Approximately one-third of the total flue gas out of the economizer enters the newly introduced bypass duct and sequentially exchanges heat in the high-temperature and low-temperature gas-water heat exchangers (HGHE and LGHE). Since the heat of the flue gas entering the main duct decreases and cannot meet the heat requirement for air preheating, two additional heat exchangers (FSHE and SSHE) are introduced for air preheating at low temperatures with the low-grade heat from the low-pressure steam extraction and the flue gas. The specifications of the main heat exchanges involved are presented in Table 2.

As given in Table 2, by employing two additional heat exchangers (SSHE and FSHE), the logarithmic mean temperature difference of the whole air preheating process is controlled above 35 °C. Particularly, by introducing the one-third of the flue gas cooled from 385 °C down to 135 °C for feedwater preheating, parts of the first, second, third, fifth and sixth-stage steam extractions are saved and continue for expansion, thus leading to remarkable performance enhancement.

This proposed system utilizes part of the waste heat from the exhausted flue gas with its temperature reducing from around 130 °C in the original design to 100 °C, due to the limitation of acid dew point of the flue gas, $t_{\mathrm{adp}}$. There are many methods to estimate the acid dew point [64]. Considering the acceptable deviation among various methods, the acid dew point is calculated here with the widely-employed method in China [65,66]:

(1) $$t_{\mathrm{adp}}=t_{\mathrm{wdp}}+\frac{\beta \cdot {\left(S_{\mathrm{ar},\mathrm{zs}}\right)}^{1/3}}{1.05^{\left(\alpha_{\mathrm{ash}}\cdot A_{\mathrm{ar},\mathrm{zs}}\right)}}$$

where $t_{\mathrm{wdp}}$ is the dew point temperature of water with the corresponding water vapor pressure of the flue gas (°C), β is the coefficient related to the excess air coefficient (β = 121) [67], $S_{\mathrm{ar},\mathrm{zs}}$ and $A_{\mathrm{ar},\mathrm{zs}}$ are the sulfur and ash content at as-received basis (kg/kcal), and finally the $\alpha_{\mathrm{ash}}$ is the fly-ash share in the fuel gas (0.9) [67]. Due to the relatively low sulfur content of the coal (approximately 0.87%), the acid dew point is estimated as 94.7 °C, which indicates that the outlet flue gas temperature of the novel WHUS can be reduced to 100 °C without serious corrosion problem.

### 2.3. The Case with ORC for Waste Heat Recovery

**Figure 2:**
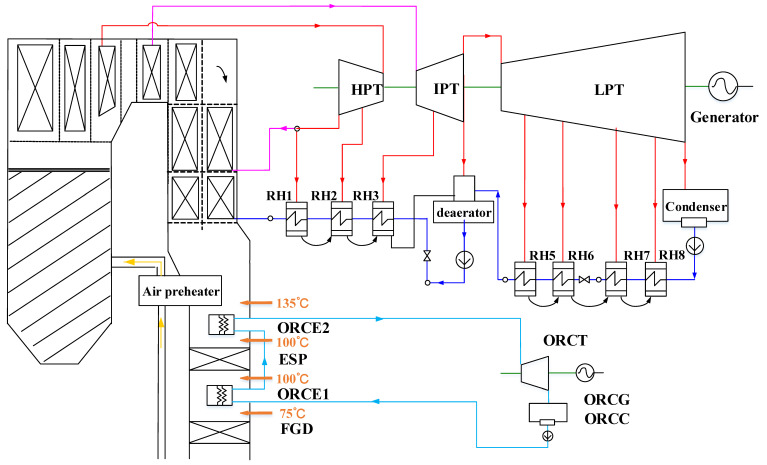
Schematic of the case with end-of-pipe waste heat utilization via ORC.

Since the in-depth boiler-turbine integration requires significant modification on the real equipment, the decision makers of existing power plants can hardly accept such a complex proposal due to potential risks and influences on the system reliability. Therefore, a simple end-of-pipe waste heat recovery system via ORC without any influence on the existing power plants (Figure 2) might be easier to be accepted.

The WHUS via ORC employs a two-stage evaporator to recover the waste heat from flue gas, which is deployed between the electrostatic precipitator (ESP) and flue-gas desulfurization unit (FGD), and between the air preheater and the ESP, respectively. Evaporator 1 recovers waste heat from the flue gas between 100 °C to 130 °C. Evaporator 2 employs fluoroplastic heat exchanger technology [68] to eliminate the corrosion problem, which allows further reduction of flue-gas temperature down to 75 °C without affecting the operating efficiency of the FGD.

The ORC system without regenerating process has been considered, since the recuperative preheating does not always lead to the maximization of net additional power output [39]. In the ORC-evaporator (ORCE), the high-pressure, sub-cooled liquid-phase working fluid is vaporized and is then expanded in the ORC-turbine (ORCT) for power generation. The expanded superheated vapor is cooled down and condensed to the saturated liquid state in the condenser (ORCC). Afterwards, the liquid-phase working fluid is pumped to the evaporator for heat absorption.

### 2.4. The Case with In-Depth Boiler-Turbine Integration and ORC

**Figure 3:**
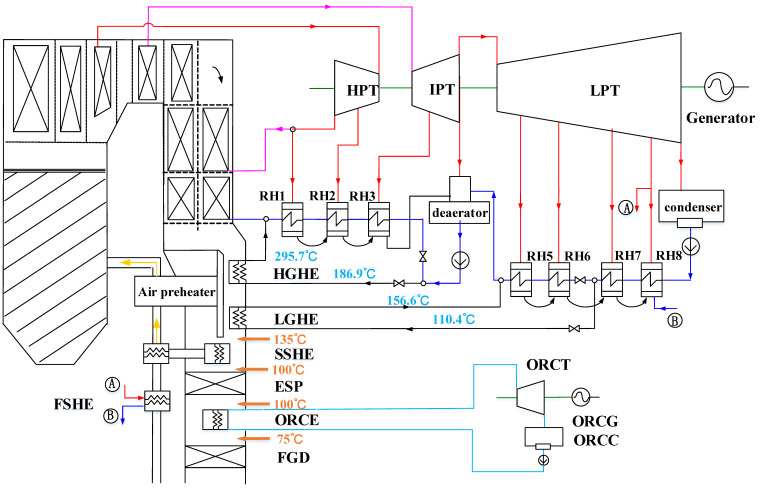
Schematic of the integrated case with both in-depth boiler-turbine integration and ORC.

The concept considering both in-depth boiler-turbine integration and ORC is mainly for more advanced, integrated plant design and aims at a further performance enhancement compared with the cases described above. The system layout is illustrated in Figure 3 with the SSHE employed for air preheating, which indicates that only one evaporator is employed for the heat absorption of the ORC, compared with Figure 2. The flue-gas temperature entering the ESP and FGD are the same as those of the ORC WHUS.

## 3. Methodology

The simulations of all concepts described in the last section are performed by Ebsilon Professional 13 [69]. Since the performance of the involved ORC is largely affected by the selected organic working fluid for given heat source, the ORC working fluid and the corresponding evaporating and condensing pressures are first selected with genetic algorithm (GA) to maximize the power generation of ORC. Then, exergetic and economic evaluation methods are described for thermo-economic comparison of the four different cases (including the reference case).

### 3.1. Working Fluid Selection of ORC

**Figure 4:**
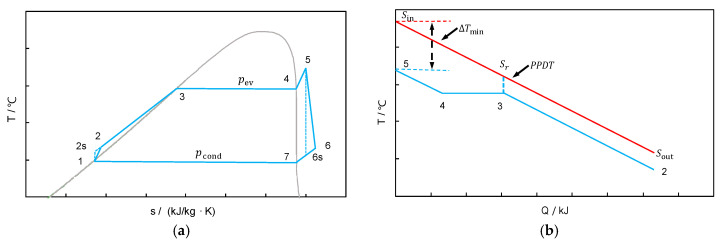
Illustrative T-s diagram (**a**); and T-Q diagram (**b**) of a sub-critical ORC ($T_{s,\mathrm{in}}$—inlet temperature of the flue gas, $T_{s,\mathrm{out}}$—outlet temperature of the flue gas, $T_{s,r}$—the temperature of the flue gas at the PPTD point, $T_{c,\mathrm{out}}$—outlet temperature of the cooling water, $T_{c,\mathrm{in}}$—inlet temperature of the flue gas, $T_{c,r}$—the temperature of the cooling water at the PPTD point, $p_{\mathrm{ev}}$—evaporating pressure, $p_{\mathrm{cond}}$—condensation pressure).

For the given heat-source temperature, a pre-selection of working fluids is performed based on [32]. Eight working fluids, i.e., R134A, Pentane, RC318, R236FA, R245FA, R123, R141B and R11, are to be evaluated. It should be mentioned that here we focus on the thermo-economic evaluation; therefore, other criteria, e.g., safety, technological feasibility and environmental harmony, are not considered. For each working fluid, the genetic algorithm is employed to optimize the two operating pressures of the ORC. The maximum allowed evaporating pressure is selected by the heat-source temperature $T_{s,\mathrm{in}}$ and the approach temperature difference $∆T_{\min}$ (see Figure 4, set as 10 °C [41]). The pinch-point temperature difference (PPTD) is set as 5 °C [41]. Note that these two temperature differences are fixed, since only thermodynamic but not economic objective is considered here.

The thermodynamic model of the sub-critical ORC is classical [39]:

(1) ORC Evaporator
  (2) $${\dot{m}}_{\mathrm{wf}}\left(h_{5}-h_{2}\right)={\dot{m}}_{s}\left(h_{s,\mathrm{in}}-h_{s,\mathrm{out}}\right)={\dot{Q}}_{\mathrm{evap}},$$
  (3) $${\dot{m}}_{\mathrm{wf}}\left(h_{5}-h_{3}\right)={\dot{m}}_{s}\left(h_{s,\mathrm{in}}-h_{s,r}\right),$$
  where the ${\dot{Q}}_{\mathrm{evap}}$ is the heat transferred in the ORC evaporator, ${\dot{m}}_{\mathrm{wf}}$ is the mass flow of the organic working fluid, ${\dot{m}}_{s}$ is the mass flow of the heat source (flue gas).
(2) ORC Turbine
  (4) $$\eta_{\mathrm{ORCT}}=\left(h_{5}-h_{6}\right)/\left(h_{5}-h_{6s}\right),$$
  (5) $${\dot{W}}_{\mathrm{ORCT}}={\dot{m}}_{\mathrm{wf}}\left(h_{5}-h_{6}\right),$$
  where the ${\dot{W}}_{\mathrm{ORCT}}$ is the power output of the ORC turbine and $\eta_{\mathrm{ORCT}}$ is the isentropic efficiency of the ORC turbine (88% [70]).
(3) ORC Condenser
  (6) $${\dot{m}}_{\mathrm{wf}}\left(h_{6}-h_{1}\right)={\dot{m}}_{c}\left(h_{c,\mathrm{out}}-h_{c,\mathrm{in}}\right)={\dot{Q}}_{\mathrm{cond}},$$
  (7) $${\dot{m}}_{\mathrm{wf}}\left(h_{7}-h_{1}\right)=\dot{m_{c}}\left(h_{c,r}-h_{c,\mathrm{in}}\right),$$
  where the ${\dot{Q}}_{\mathrm{cond}}$ is the heat transferred in the ORC condenser and ${\dot{m}}_{c}$ is the mass flow of the cooling water.
(4) ORC Pump
  (8) $$\eta_{\mathrm{ORCP}}=\left(h_{2s}-h_{1}\right)/\left(h_{2}-h_{1}\right),$$
  (9) $${\dot{W}}_{\mathrm{ORCP}}={\dot{m}}_{\mathrm{wf}}\left(h_{2}-h_{1}\right),$$
  where the ${\dot{W}}_{\mathrm{ORCP}}$ is the power consumed by the ORC pump and $\eta_{\mathrm{ORCP}}$ is the isentropic efficiency of the ORC pump (80% [70]).

The pressure drops as well as heat losses are neglected for all ORC equipment [39]. The net power output (${\dot{W}}_{\mathrm{ORC}}$) of the ORC is given as below:

(10) $${\dot{W}}_{\mathrm{ORC}}={\dot{W}}_{\mathrm{ORCT}}-{\dot{W}}_{\mathrm{ORCP}}-{\dot{W}}_{\mathrm{ORCC}}$$

where ${\dot{W}}_{\mathrm{ORCC}}$ is the power consumed by the recycle cooling water pump, which is calculated with the inlet temperature of the cooling water of 20 °C.

**Figure 5:**
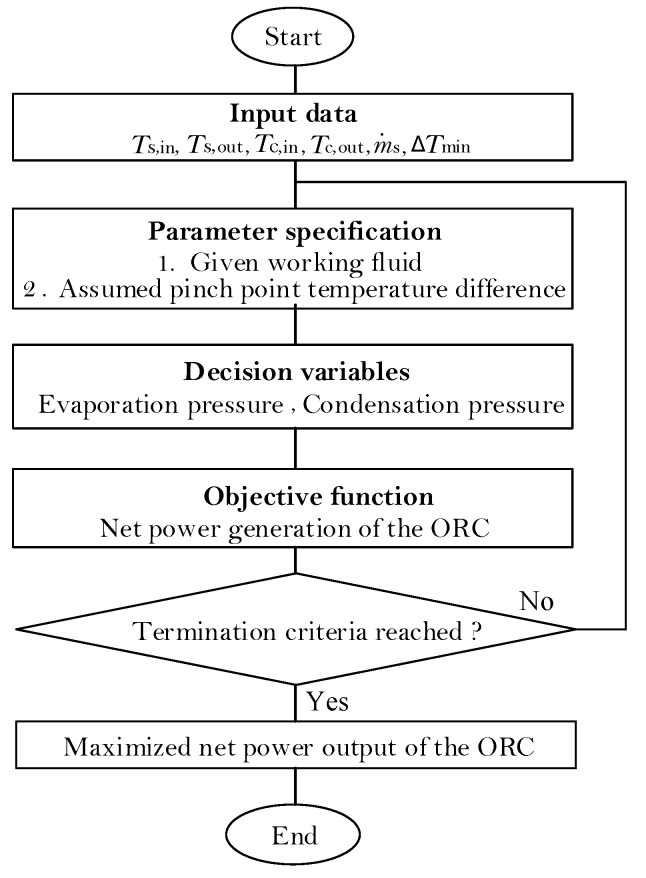
Simplified flow chart of the optimization procedure.

The optimization procedure for working-fluid selection and the corresponding operating variables is illustrated in Figure 5.

### 3.2. Exergy Analysis

Exergy analysis is employed in this paper to explore the difference in the energy utilization of the four cases. Exergy analysis can identify the sources and the magnitudes of exergy destructions and losses of energy systems [71,72,73], thus highlighting the processes with the largest exergy destruction or loss as the key components or processes for potential system improvement [1,74,75,76]. Note that the exergy losses do not occur at a single component level but only at the overall system level, when the system is interacting with the environment via vent streams.

The exergy balance of the component k can be calculated as following formulation [77]:

(11) $${\dot{E}}_{D,k}={\dot{E}}_{F,k}-{\dot{E}}_{P,k},$$

where the symbols ${\dot{E}}_{F,k}$ ${\dot{E}}_{P,k}$ ${\dot{E}}_{D,k}$ represent the fuel exergy, product exergy and exergy destruction, respectively. With the clear definitions of fuel exergy, product exergy, the exergy efficiency of a productive component k can be expressed as:

(12) $$\varepsilon_{k}={\dot{E}}_{P,k}/{\dot{E}}_{F,k}=1-{\dot{E}}_{D,k}/{\dot{E}}_{F,k}.$$

The exergy balance equation of the overall system can be formulated as below:

(13) $${\dot{E}}_{F,\mathrm{tot}}={\dot{E}}_{P,\mathrm{tot}}+\sum {\dot{E}}_{D,k}+{\dot{E}}_{L,\mathrm{tot}},$$

with the total exergy efficiency of the overall system defined as

(14) $$\varepsilon_{\mathrm{tot}}={\dot{E}}_{P,\mathrm{tot}}/{\dot{E}}_{F,\mathrm{tot}}.$$

The detailed calculation procedure of chemical and physical exergies about various material flows, work and heat streams can be referred in the literature [78]. The chemical exergy of coal also calculated as the product of the higher heating value (HHV) with a constant, usually 1.02 [77]. In addition, the reference environment for chemical exergy calculation is defined at 298.15 K and 1.098 bar [77].

### 3.3. Economic Analysis

Basic economic assumptions employed are listed as follows: (1) The on-grid power tariff is set as 0.061 USD/kWh and the annual full-load operating hours (e.g., the annual utilization hours) are assumed as 5000 h, which indicates that the average operating load factor is below 100% due to the frequent participation in peak load regulation of large-scale coal-fired power units; (2) The auxiliary cost is fixed as 15% of the bare module cost for all components [79], which indicates a common total module factor ($f_{\mathrm{TM}}$) of 0.15; (3) The operation and maintenance cost ($C_{O\&M}$) accounts for 4% of the annualized total capital investment (TCI) [24,80]; (4) The exchange rate of RMB to U.S. dollar is set as 6.25 CNY/USD.

#### 3.3.1. Estimation of Purchased Equipment Cost

The total capital investment of a component can be estimated based on its purchased equipment cost (PEC) [26,81], which is employed for the estimation of the corresponding bare module cost ($C_{\mathrm{BM}}$) by further considering a bare module factor ($f_{\mathrm{BM}}$) due to the selection of material, the operating temperature and pressure, etc. Thus, for this retrofit problem, the total capital investment cost can be calculated as follows:

(15) $$TCI=\sum_{i}C_{\mathrm{TM},i}=\sum_{i}C_{\mathrm{BM},i}\left(1+f_{\mathrm{TM}}\right)=\sum_{i}PEC_{i}f_{\mathrm{BM}}\left(1+f_{\mathrm{TM}}\right).$$

The purchased equipment cost of a component can be estimated by a simple widely-used scaling method [81,82], if not many investment data are available for the considered component type:

(16) $$PEC_{i}=PEC_{\mathrm{ref}}{\left(\frac{S}{S_{\mathrm{ref}}}\right)}^{\alpha }$$

where the symbols S and α denote the selected sizing parameter and scaling factor, respectively, while the $PEC_{\mathrm{rerf}}$ and $S_{\mathrm{ref}}$ represent the reference capital investment and scaling parameter of the reference component, respectively. If there have been many investment data available for different sizes of the investigated component, the interested purchased equipment cost can be simply regressed with respect to the sizing parameters. For example, the PEC of heat exchangers (HEX), pump and turbine, can be estimated as follows [83,84]:

(17) $$\log PEC_{\mathrm{HEX}}=K_{1}+K_{2}\log A+K_{3}{\left(\log A\right)}^{2}$$

(18) $$\log PEC_{\mathrm{PUMP}}=K_{1}+K_{2}\log W+K_{3}{\left(\log W\right)}^{2}$$

(19) $$\log PEC_{\mathrm{TURBINE}}=K_{1}+K_{2}\log W+K_{3}{\left(\log W\right)}^{2}$$

where the co-efficients *K*_1_–*K*_3_ can be obtained by the regression and are 3.853, 0.424 and 0 for heat exchangers, 3.579, 0.321 and 0.003 for the pump, and 3.514, 0.598 and 0 for turbine [83]. For heat exchangers, pump and turbine, the sizing parameters are taken as area and shaft work, respectively. The bare module factor ($f_{\mathrm{BM}}$) is generally a function of the material factor ($f_{M}$), the pressure factor ($f_{P}$), the temperature factor ($f_{T}$) as well as the actualization factor ($f_{A}$) as follows [82]:

$$f_{\mathrm{BM}}=ℱ\left(f_{M},f_{P},f_{T},f_{A}\right)$$

The actualization factor is employed to convert the capital cost in the reference year to the actualization year, which can be based on different cost index, e.g., Marshall and Swift index (MS) and Chemical Engineering Plant Cost Index (CEPCI) [83].

For the system with only in-depth boiler-turbine integration, the TCI for retrofitting the original power plant mainly consists of the capital investment of the newly-added exchangers (HEX). The related capital investment is estimated based on the simple method (Equation (16)) with the reference cost data and all necessary factors given in Table 3. The bare module cost, in this case, is a product of all factors: $f_{\mathrm{BM}}=f_{M}\;f_{P}\;f_{T}\;f_{A}$.

For the case with only the ORC for waste heat recovery, the total capital investment comes from the evaporate, condenser, pump, and turbine, since the contribution of the fluid cost to the total investment cost is negligible, as shown in Refs. [34,42,83]. The costs of the evaporator and condenser are estimated by Equation (17) with the bare module factor obtained by [85]:

(20) $$f_{\mathrm{BM}}=\left(B_{1}+B_{2}f_{M}f_{P}\right)$$

and the pressure factor formulated as

(21) $$\log F_{P}=C_{1}+C_{2}\log p+C_{3}{\left(\log p\right)}^{2}$$

The coefficients $B_{1}$, $B_{2}$, $C_{1}$, $C_{2}$, $C_{3}$ are 1.53, 1.27, 0, 0, and 0 for turbines and heat exchangers, 1.8, 1.51, 0.168, 0.348 and 0.484 for pumps, respectively [83]. The costs of pumps and turbines are computed via Equations (18) and (19).

For the case with both ORC and in-depth boiler-turbine integration, the total capital investment is comprised of all newly-added heat exchangers (including the gas-air HEX, water-air HEX, ORC evaporator and condenser), pumps and turbines with the equations and coefficients listed above. 

With the total capital investment calculated for a specific retrofit, the annualized investment capital cost ($C_{\mathrm{TCI}}$) can be calculated as [81,82,86]:

(22) $$C_{\mathrm{TCI}}=TCI\frac{i{\left(1+n\right)}^{i}}{{\left(1+n\right)}^{i}-1}$$

where the symbol i refers to the interest rate per year (8%), while the *n* represents the system lifespan (20 years).

#### 3.3.2. Economic Performance Indicators

The feasibility of the three different retrofit concepts is further evaluated by an economic benefit, which is represented by the net annual revenue (NAR):

(23) $$NAR=EAI-C_{\mathrm{TCI}}-C_{O\&M}$$

where the EAI stands for the additional income per year of the overall plant after the specific retrofit:

(24) $$EAI=∆P_{\mathrm{net}}\tau C_{e}$$

where the $∆P_{\mathrm{net}}$ represents the net additional power output for the full-load operation of the plant after the retrofit, the τ is the equivalent full-load operation hours per year (5000 h as mentioned above), and the $C_{e}$ stands for the tariff of the electricity sent to the grid.

In addition, the pay-back period (Pt) for the total capital investment, a vital indicator to show the economic feasibility of a technology, is calculated as follows [87]:

(25) $$\mathrm{Pt}=-\log_{\left(1+i\right)}\left(1-\frac{TCI\cdot i}{EAI}\right)$$

## 4. Results and Discussion

The three different retrofit concepts are comprehensively evaluated and discussed as follows: the selection of working fluid of the ORC, the comparison of the thermodynamic performances and the comparison of economic performances. 

### 4.1. The Selection of ORC Working Fluid

Eight working fluids are screened in terms of the net additional power output with respect to two operating variables: the pressures of evaporation and condensation. The optimal operating conditions of each working fluid are given in Table 4 for the system retrofitted with both the in-depth boiler-turbine integration and ORC.

**Figure 6:**
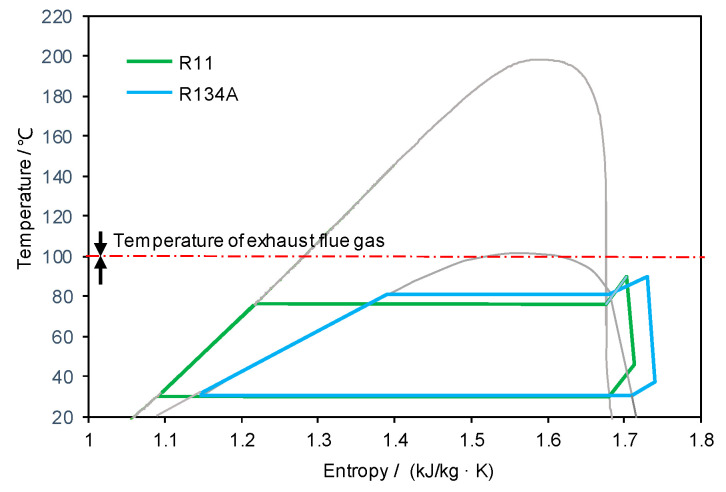
T-s diagrams of the ORCs with R11 and R134A.

From the perspective of the turbine output power, the R134A is the optimal fluid with 3.07 MW total power generated, followed by the R11 (2.93 MW). The T-s diagrams of both R134A- and R11-based ORC are illustrated in Figure 6 for the thermodynamic comparison of the two cycles. It is also found in Table 4 that the power-generation difference between different working fluids is not significant. Considering the pumping work, the fluid R11 achieves the largest net power generation (2.52 MW) with the corresponding evaporation pressure of 0.48 MPa and the condensation pressure of 0.13 MPa; while the fluid R134A requires higher pump work due to a much higher evaporation pressure (2.69 MPa, which leads to a net power output of 2.41 MW). Therefore, we consider the fluid R11 as the optimal. All the calculations and comparisons related to ORC are based on this fluid and its corresponding optimal working conditions.

### 4.2. Exergy Analysis

Compared with the original net power output (993.8 MW), an additional 23.99 MW, 6.49 MW, and 26.51 MW net power is produced with only in-depth boiler-turbine integration, only the ORC, and both the ORC and boiler-turbine integration. The in-depth boiler-turbine integration contributes the most to boost the power output, since the temperature level of flue gas up to 385 °C has been considered for heat integration. The same decrease in the temperature difference of heat transfer, which is the driving force of the increase in power output, allows much more heat available for power generation, due to the wide temperature range of heat source. The additional work resulted from the utilization of the waste heat of flue gas from 135 °C down to 75 °C itself is limited no matter how small the heat-transfer temperature difference is reached. However, the ORC can still be employed with the in-depth boiler-turbine integration to reach a high-level waste heat recovery of the overall system.

**Figure 7:**
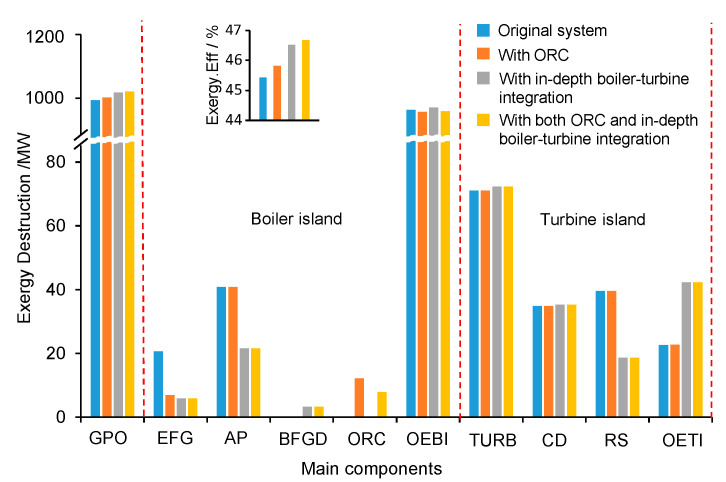
Exergy destruction distribution of main components for different integration concepts (GPO—gross power output, EFG—exhaust flue gas, AP—air preheating, BFGD—bypass flue gas duct, ORC—organic Rankine cycle, OEBI—other equipment of boiler island, TURB—Turbine, CD—condenser, RS—regenerative system, OETI—other equipment of turbine island).

The exergy analysis provides more insights on how each new integration concept eventually improves the system performance, with the comparison of exergy destructions within different components for different concepts in Figure 7. Due to the increased power output with the same amount of fuel consumption, the overall exergy efficiency of the system has been increased by 0.39 percentage points when only with ORC, 1.1 percentage point when only with the in-depth boiler-turbine integration, and 1.25 percentage point when with both concepts. It is also found in the in-depth turbine-boiler integration system that the reduction of the exergy destruction within the boiler subsystem reaches 27.75 MW, far larger than that within the turbine subsystem, 0.48 MW. Therefore, it is concluded that the energy-saving improvement is mainly contributed by the performance enhancement of the boiler subsystem.

More specifically, the decrease in exergy destruction within the boiler island is mainly due to the improvement of air preheating (AP), which reduces its exergy destruction from 40.77 MW to 21.54 MW. This is mainly because of the utilization of the two-stage air preheater exchangers as well as the two-stage gas-water heater exchangers in the bypass flue gas duct (BFGD). The heat transfer temperature difference of the concept with both ORC and in-depth boiler-turbine integration decreases significantly due to the better temperature match of the hot and cold fluids after dividing the air preheating process into three parts. Although with the bypass flue-gas duct, the exergy destructions within the high-temperature gas-water heat exchangers and low-temperature gas-water heat exchangers increase by 2.38 and 0.86 MW, the system recovers 26.46 MW exergy from the flue gas going through the bypass flue-gas duct. Therefore, the exergy destruction within the boiler island can be saved up to 27.75 MW when with both ORC and in-depth boiler-turbine integration, and the exergy losses from flue gas (EFG) are largely reduced as well.

For the turbine island, the variations of the exergy destruction within the turbine (TURB) and condenser (CD) are rather small (only 1.66 MW), compared to that within the regenerative system (RS). The beauty of the in-depth boiler-turbine integration is that the new layout promotes some feedwater and condensate water acquiring heat from the flue gas via the two-stage gas-water heat exchangers configured in the bypass flue gas duct in the boiler island, which generally results in a significant reduction of the heat needed from the steam extractions and a boost of power output due to this suppress of steam extractions. The exergy destruction within the RS is reduced remarkably by 21.87 MW. However, the additional heat exchangers require much power to overcome the flow resistance, the exergy destruction within the other parts of the turbine (OETL), including the valves and pipeline, etc., increases also dramatically due to the pure power consumption, which eventually leads to an increase in total exergy destruction within the turbine island by 0.48 MW, compared to that of the original system.

For the ORC system, the exergy destructions are 13.26 MW and 8.19 WM for the systems with only the ORC and with both the ORC and the in-depth turbine-boiler interaction. The difference is mainly due to the different layouts of the ORC systems in both concepts, since there is two-stage evaporator for the former while one-stage evaporator for the latter.

**Figure 8:**
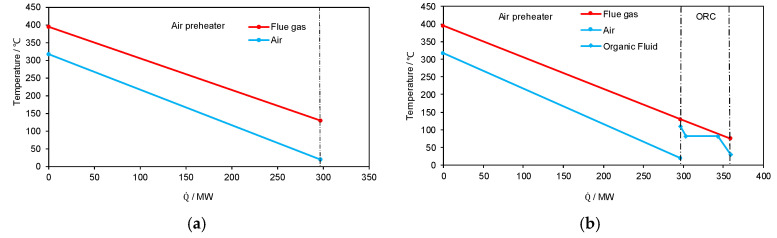

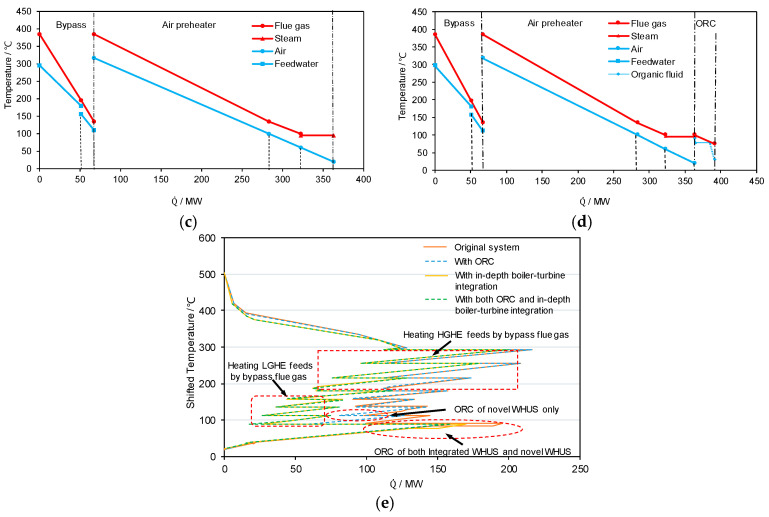
Thermodynamic insights on the system improvement of different integration concepts. (**a**) Original System; (**b**) The concept with only the ORC; (**c**) The concept with only the in-depth boiler-turbine integration; (**d**) The concept with both the ORC and in-depth boiler-turbine integration; (**e**) Grand composite curves of the varied parts of all four concepts.

More thermodynamic insights are given in Figure 8 to further illustrate how the system is improved with different integration concepts. With only one-stage of air-preheating, the mass flowrate and temperature of the air at an intermediate stage cannot be flexibly adjusted, which means the slopes of the two straight lines in Figure 8a are fixed. The upper-terminal temperature difference and the exergy destruction of the air preheater can be reduced by an increased air-preheater area; however, the increase in the final air-preheating temperature will increase the temperature of combustion, thus increasing the exergy destruction within the remaining boiler subsystem. This eventually increases the inlet temperature of the flue gas entering the air preheater. Therefore, increasing the air-preheater area is not a way of reasonably utilizing the heat at the boiler cold end. With the integration of only ORC (Figure 8b), there is no modification of the boiler subsystem at all. Only the heat of the flue gas at a temperature lower than 100 °C is utilized, which brings additional benefits without affecting the existing plant. However, the benefit is largely limited by the available heat extracted from the flue gas at a low temperature until the due temperature.

The essence of the in-depth boiler-turbine integration is given in Figure 8c. With the splitting of the hot and cold fluids, the slopes of the Q-T profiles are in fact readily adjusted, which gives a much better opportunity for establishing better matches of temperature levels of heat transfer. Particularly, the temperature difference of the bypass flue gas duct can be reduced largely but still beyond the industrial minimum temperature difference usually employed. In addition, by the splitting of flue gas and multiple stages of air preheating, a smaller temperature difference for heat transfer can be successfully achieved without modifying the system too much. Further, coupling with the ORC system for ultra-low temperature waste heat allows for an even larger benefit can be achieved based on the in-depth boiler-turbine integration.

The grand composite curves of the varied parts of the four plants (Figure 8e) offers a much more straightforward comparison of the benefits from different concepts. With the in-depth boiler-turbine integration, the energy pocket enclosed by the flue gas is reduced significantly, which means more heat is extracted for better utilization (power generation). The pocket reduction realized by the ORC is also illustrated, although limited.

### 4.3. Economic Analysis

The capital investment costs of the newly-added components of the three concepts are listed in Table 5. The additional capital investment cost introduced by the new components of in-depth turbine-boiler integration is 5.327 USD million, more than that of the ORC (3.951 USD million). The turbine and heat exchangers of the ORC contribute significantly to the total capital investment, leading to the fact that the potential of reducing the turbine cost and heat exchangers expense should be investigated. The integrated system with ORC and in-depth boiler-turbine integration needs the largest capital investment, 7.550 USD million, slightly less than the sum of both, due to the cost reduction of the equipment for the integrated system.

The economic feasibility of the three improved systems is further investigated by various economic indicators listed in Table 6. The EAI of the in-depth turbine-boiler integration is 7.317 million USD, three times more than that of the system with only the ORC (1.979 million USD). However, too many newly-added components, together with the high capital investment, increase the system reliability and complexity, which further results in an increased operation and maintenance cost. The EAI of the concept with only the ORC is 1.979 million USD with a C_TCI_ of 1.462 million USD. Therefore, the system with only the ORC is less suggested to be applied solely at the current technology level. The NAR of the integrated system is 4.990 million USD, indicating its excellent economic performance and better implementation potential. The investment-payback period of the system with only the ORC is almost 2.26 years to recover the total investment, while the system with the in-depth boiler-turbine integration only needs 0.78 years. The single implementation of the ORC is less economically viable since large capital investment is needed for less gain in power output. This also indicates that the ORC technology is still quite expensive, and its economic competitiveness needs to be further enhanced by the technology developers. By integrating both concepts, the integrated system has an intermediate payback time of 1.01 years, which is still acceptable and may be helpful to promote the development and to reduce the cost of the ORC technology.

## 5. Conclusions

In this paper, as a follow-up to our previous research, we proposed a novel waste heat recovery system based on a comprehensive understanding of the ORC and in-depth system integration. Then, we comparatively evaluated three integration concepts of the large-scale coal-fired power plant to better utilize the heat at an overall system level, particularly that at intermediate and low grade. The three concepts employ only ORC, only in-depth boiler-turbine integration, and both, respectively. A comprehensive and comparative evaluation of these three concepts is performed in terms of thermodynamic and economic performances to evaluate their feasibility and competitiveness. The major conclusions are as follows:

For the in-depth boiler-turbine integration system, by splitting the flue gas, the heat capacities of the hot streams can be adjusted to have a better match with available cold streams, thus fulfilling much better overall heat integration. Therefore, the multi-stage air preheating is made possible and significantly reduces the heat-transfer temperature difference. The exergy destruction of the air preheating process is reduced by 19.23 MW. By introducing this part of flue-gas heat to the turbine system, the steam extractions can be largely suppressed, thus largely boosting the power output. The in-depth boiler-turbine integration demonstrated an additional power output of 23.99 MW and high economic competitiveness (with a payback time of only 0.78 years).

The selection of the working fluid with respect to available heat sources is important to maximize the ORC performance. The best ORC working fluid found out of eight for the given flue gas temperature (100 °C) is R11, which can be fully vaporized at such a low temperature and, therefore, offer better work ability. With the utilization of its latent heat, the mass flowrate of R11 in the ORC is much lower than those of the other working fluids. With R11-based ORC, the exergy loss from the exhausted flue gas is reduced from 20.69 MW to 5.90 MW, which results in an additional power output of 6.49 MW.

Compared with in-depth boiler-turbine integration, the waste heat recovery via ORC only delivers limited effect to further increase the power output; however, its high investment cost and lower payback time make its single application hardly acceptable in a near term. However, by coupling the ORC with in-depth boiler-turbine integration, the payback time can be largely reduced, indicating a better choice to promote the large-scale deployment of ORC and cost reduction.

## Figures and Tables

**Table 1:** Major stream data for the turbine heat rate acceptance (THA) condition.

Item	Unit	RH1	RH2	RH3	DEA	RH5	RH6	RH7	RH8
Temperature of steam extraction	°C	421.8	339.6	505.4	396.5	326.0	252.9	170.1	95.7
Pressure of steam extraction	MPa	8.13	4.54	2.28	1.11	0.64	0.36	0.17	0.08
Temperature of outgoing feedwater	°C	295.7	256.1	217.6	186.9	156.6	135.2	110.4	87.9
Pressure of outgoing feedwater	MPa	27.87	27.88	27.89	1.04	1.05	1.06	1.07	1.08
Temperature of incoming feedwater	°C	256.1	217.6	186.9	156.6	135.2	110.4	87.9	38.6
Pressure of the incoming feedwater	MPa	27.88	27.89	27.90	1.05	1.06	1.07	1.08	1.09
Temperature of the drainage	°C	261.7	223.2	192.5	—	140.8	116.0	93.5	44.2

**Table 2:** Specifications of the main heat exchangers involved.

Item	Unit	HGHE	LGHE	FSHE	SSHE
Inlet temperature of flue gas	°C	385	196	—	135
Outlet temperature of flue gas	°C	196	135	—	100
Inlet temperature of water/steam	°C	186.9	110.4	95.4(1 *)	—
Outlet temperature of water/steam	°C	295.7	156.6	95.4(0 *)	—
Inlet temperature of air	°C	—	—	20	60
Outlet temperature of air	°C	—	—	60	100
Logarithmic mean temperature difference	°C	35.13	31.40	46.29	37.03
Heat exchanger area	m^2^	30,847	12,895	15,061	29,576

* Steam quality: dryness.

**Table 3:** Reference cost data for newly-added heat exchangers.

Component	Reference Cost ($PEC_{\mathrm{ref}}$, Million $)	Sizing Parameter ($S_{\mathrm{ref}}$, m^2^)	Scaling Factor (α)	$f_{M}$	$f_{P}$	$f_{T}$	$f_{A}$
Heater	0.693	13,149	0.68	1.00	1.00	1.20	1.08
Air Preheater	7.07	421,963	0.68	1.00	1.00	0.95	1.05

**Table 4:** Optimal operating conditions and gains of the ORC with various working fluids.

Fluid	Evaporation Pressure (MPa)	Condensation Pressure (MPa)	Mass Flowrate (kg/s)	Pump Work (MW)	Turbine Output (MW)	ORC Net Power Output (MW)
R134A	2.69	0.79	139.90	0.67	3.07	2.41
Pentane	0.34	0.08	60.51	0.39	2.82	2.43
RC318	1.41	0.37	199.05	0.55	2.79	2.24
R236FA	1.25	0.32	153.11	0.51	2.89	2.38
R245FA	0.76	0.18	119.04	0.46	2.90	2.45
R123	0.46	0.11	134.74	0.44	2.92	2.48
R141B	0.38	0.09	104.59	0.40	2.91	2.51
R11	0.48	0.13	132.57	0.41	2.93	2.52

**Table 5:** The capital investment cost of the newly-added equipment (unit: million USD).

Item	In-Depth Boiler-Turbine Integration	Only ORC	Integrated System
High-temperature gas-water heat exchanger	1.604	—	1.604
Low-temperature gas-water heat exchanger	0.886	—	0.886
Second-stage heat exchanger	0.985	—	0.985
First-stage heat exchanger	1.157	—	1.157
Auxiliary equipments	0.695	—	0.695
Organic Rankine cycle evaporator	—	1.035	0.661
Organic Rankine cycle turbine	—	1.429	0.646
Organic Rankine cycle condenser	—	1.132	0.720
Organic Rankine cycle pump	—	0.355	0.196
Total investment cost	5.327	3.951	7.550

**Table 6:** Revenue analysis (units: MW, million USD and year).

Item	In-Depth Boiler-Turbine Integration	Only ORC	Integrated System
Net additional power output	23.99	6.49	26.51
Extra annual income (EAI)	7.317	1.979	8.087
Annualized investment capital cost (C_TCI_)	1.971	1.462	2.794
Operation & maintenance cost (C_O&M_)	0.213	0.158	0.302
Net annual revenue (NAR)	5.132	0.358	4.990
Dynamic investment payback period (Pt)	0.78	2.26	1.01

## Abbreviations

WHUS	Waste heat recovery system
ORC	Organic Rankine Cycle
PPTD	Pinch Point Temperature Difference
EPC	Electricity Production Cost
RH	Regenerative heater
LTEs	Low-temperature economizers
THA	The design condition
HPT	High-pressure turbine
IPT	Intermediate-pressure turbine
LPT	Low-pressure turbine
HGHE	High-Temperature Gas-water heat exchanger
LGHE	Low-Temperature Gas-water heat exchanger
FSHE	First-stage heat exchanger
SSHE	Second-stage heat exchanger
ESP	Electrostatic precipitator
FGD	Flue gas desulfurization
NAR	The net annual revenue
ORCE	Organic Rankine Cycle Evaporator
ORCT	Organic Rankine Cycle Turbine
ORCC	Organic Rankine Cycle Condenser
HHV	Higher heating value
O&M	Operation and maintenance
CEPCI	Chemical Engineering Plant Cost Index
Pt	Dynamic investment pay-back period
GPO	Gross power output
EFG	Exhaust Flue Gas
AP	Air Preheating
BFGD	Bypass Flue Gas Duct
OEBI	Other Equipment of Boiler Island
TURB	Turbine
CD	Condenser
RS	Regenerative System
OETI	Other Equipment of Turbine Island
TIC	Total investment capital
EAI	Extra annual income
**Symbols**	
$t_{\mathrm{adp}}$	Acid dew point, °C
$t_{\mathrm{wdp}}$	Dew point temperature of water, °C
β	Coefficient related to the excess air coefficient
$S_{\mathrm{ar},\mathrm{zs}}$	Sulfur content at as-received basis, kg/kcal
$A_{\mathrm{ar},\mathrm{zs}}$	Ash content at as-received basis, kg/kcal
$\alpha_{\mathrm{ash}}$	Fly-ash share in the fuel gas
${\dot{Q}}_{\mathrm{evap}}$	Heat transferred in the ORC evaporator
${\dot{m}}_{\mathrm{wf}}$	Mass flow of the organic working fluid
${\dot{m}}_{s}$	Mass flow of the heat source (flue gas)
${\dot{W}}_{\mathrm{ORCT}}$	Power output of the ORC turbine
$\eta_{\mathrm{ORCT}}$	Isentropic efficiency of the ORC turbine
${\dot{Q}}_{\mathrm{cond}}$	Heat transferred in the ORC condenser
${\dot{m}}_{c}$	Mass flow of the cooling water
${\dot{W}}_{\mathrm{ORCP}}$	Power consumed by the ORC pump, MW
$\eta_{\mathrm{ORCP}}$	Isentropic efficiency of the ORC pump
${\dot{W}}_{\mathrm{ORC}}$	The net power output of ORC system, MW
${\dot{W}}_{\mathrm{ORCC}}$	Power consumed by the recycle cooling water pump, MW
$T_{s,\mathrm{in}}$	The inlet temperature of the flue gas, °C
$T_{s,\mathrm{out}}$	The outlet temperature of the flue gas, °C
$T_{s,r}$	The temperature of the flue gas at the PPTD point, °C
$T_{c,\mathrm{out}}$	The outlet temperature of the cooling water, °C
$T_{c,\mathrm{in}}$	The inlet temperature of the flue gas, °C
$T_{c,r}$	The temperature of the cooling water at the PPTD point, °C
$p_{\mathrm{ev}}$	Evaporating pressure, MPa
$p_{\mathrm{cond}}$	Condensation pressure, MPa
${\dot{E}}_{F,k}$	Fuel exergy, kJ/kg
${\dot{E}}_{P,k}$	Product exergy, kJ/kg
${\dot{E}}_{D,k}$	Exergy destruction, exergy loss
$\varepsilon_{\mathrm{tot}}$	The total exergy efficiency
$C_{\mathrm{BM}}$	Bare module cost, million USD
$f_{\mathrm{BM}}$	Bare module factor
*S*	The size parameters of equipment
α	Scale factor
$PEC_{\mathrm{rerf}}$	Reference capital investment of the reference component, million USD
$S_{\mathrm{ref}}$	Scaling parameter of the reference component
*K*	Coefficients for the cost evaluation
$f_{M}$	The Material factor
$f_{P}$	The Pressure factor
$f_{T}$	The Temperature factor
$f_{A}$	The actualization factor
$∆P_{\mathrm{net}}\;\;$	Net additional power output, MW
$C_{e}$	On-grid power tariff, USD/kWh
τ	The equivalent full-load operation hours per year, h
i	Fraction interest rate per year, %
n	Number of years
